# Use of Artificial Intelligence in Triage in Hospital Emergency Departments: A Scoping Review

**DOI:** 10.7759/cureus.59906

**Published:** 2024-05-08

**Authors:** Samantha Tyler, Matthew Olis, Nicole Aust, Love Patel, Leah Simon, Catherine Triantafyllidis, Vijay Patel, Dong Won Lee, Brendan Ginsberg, Hiba Ahmad, Robin J Jacobs

**Affiliations:** 1 Medicine, Dr. Kiran C. Patel College of Osteopathic Medicine, Nova Southeastern University, Fort Lauderdale, USA

**Keywords:** ai, clinical decision support system, emergency department, language learning model, machine learning algorithms, machine learning (ml), triage care

## Abstract

The integration of artificial intelligence (AI) and machine learning (ML) in healthcare has become a major point of interest and raises the question of its impact on the emergency department (ED) triaging process. AI’s capacity to emulate human cognitive processes coupled with advancements in computing has shown positive outcomes in various aspects of healthcare but little is known about the use of AI in triaging patients in ED. AI algorithms may allow for earlier diagnosis and intervention; however, overconfident answers may present dangers to patients. The purpose of this review was to explore comprehensively recently published literature regarding the effect of AI and ML in ED triage and identify research gaps. A systemized search was conducted in September 2023 using the electronic databases EMBASE, Ovid MEDLINE, and Web of Science. To meet inclusion criteria, articles had to be peer-reviewed, written in English, and based on primary data research studies published in US journals 2013-2023. Other criteria included 1) studies with patients needing to be admitted to hospital EDs, 2) AI must have been used when triaging a patient, and 3) patient outcomes must be represented. The search was conducted using controlled descriptors from the Medical Subject Headings (MeSH) that included the terms “artificial intelligence” OR “machine learning” AND “emergency ward” OR “emergency care” OR “emergency department” OR “emergency room” AND “patient triage” OR “triage” OR “triaging.” The search initially identified 1,142 citations. After a rigorous, systemized screening process and critical appraisal of the evidence, 29 studies were selected for the final review. The findings indicated that 1) ML models consistently demonstrated superior discrimination abilities compared to conventional triage systems, 2) the integration of AI into the triage process yielded significant enhancements in predictive accuracy, disease identification, and risk assessment, 3) ML accurately determined the necessity of hospitalization for patients requiring urgent attention, and 4) ML improved resource allocation and quality of patient care, including predicting length of stay. The suggested superiority of ML models in prioritizing patients in the ED holds the potential to redefine triage precision.

## Introduction and background

In the US, there are approximately 131 million hospital emergency department (ED) visits annually, 19 million of which lead to hospital admissions [1]. There has been a marked increase in the number of ED visits due to the uptick in nonurgent visits, recurrent patients, staff shortages, and reductions of downstream beds [2]. These statistics are not only a significant source of stress for healthcare professionals but also lead to negative effects on patient mortality, complications, walkouts, and length of stay. Many of the solutions proposed to address the increase in patient volume focus on improving patient workflow but have not adequately improved these negative outcomes [2]. Examples of solutions to improve patient workflow include extended general practice hours, earlier physician assessment and physician-led triage, increased ED staff, and increased ED bed numbers [3]. Other solutions include addressing avoidable ED attendances by diverting patients where they could be more adequately treated, preventing the progression of disease with earlier intervention or better management of their condition, and preventing visits in which the patient did not require any clinical care [4]. These solutions are aimed at addressing patient volume rather than improving patient outcomes through efficient and proper triage methods.

Artificial intelligence (AI) and machine learning (ML) have become novel topics for triage in EDs due to several key factors, including 1) the possibility of enhanced decision-making by analyzing vast amounts of patient data quickly and accurately; 2) efficiency and speed may be increased in often crowded, fast-paced EDs by using AI-powered triage tools to streamline the process by quickly assessing patient symptoms, medical history, and other relevant factors; 3) ML algorithms can be trained on historical data to predict the severity of patients' conditions and identify those at risk of deteriorating, which can help ED staff proactively manage patient flow, allocate resources efficiently, and respond to emergencies more effectively; and 4) advanced data analytics can enable EDs to triage patients with a more comprehensive understanding of their health status. Moreover, AI may help decrease inconsistency in human decision-making caused by stress, fatigue, and personal biases [3-6]. 

Recently, new developments with AI have been incorporated into healthcare to improve efficiency, accuracy, workflow, and costs, and provide better healthcare overall [5,6]. AI is best defined as a machine capable of imitating intelligent human cognitive processes and behavior [6]. Human reasoning capability along with computing speed, storage capability, and interconnectedness is what gives AI the unique ability to formulate solutions to complex and large-scale problems [7]. Medical AI has two main branches including virtual (ML or deep learning) and physical (medical devices). The virtual component is represented by mathematical algorithms that can be unsupervised (find patterns), supervised (previous examples), and reinforced (sequence or rewards and punishments) [8]. AI algorithms that are capable of rapidly analyzing and interpreting a large amount of patient data are expected to be able to identify patterns and trends that may not be immediately recognized by providers, allowing for earlier diagnosis and intervention, and contributing to healthcare operation efficiency and patient flow [9,10]. Physical medical AI is related to medical devices or robots that can assist in providing care or surgeries [8].

AI has become part of the foundation of system-wide improvement in emergency services through improving patient diagnosis, analyzing imaging, assisting in medical decision-making, predicting clinical outcomes, resource planning, and patient monitoring system integration [11]. Past studies have shown that AI can be beneficial in addressing ED overcrowding, diagnostic modeling, and rapid and effective triaging based on severity grades [12,13]. Effective triaging of many patients by the triage nurse upon admission to the ED is an essential role to ensure timely treatment based on the level of urgency. Triaging is generally done with five-level triage scales, relying on the subjective judgment of the triage nurse, which is inherently subject to variation both within the ED and among hospital systems due to a wide array of influences like training, hospital policies, and experience of the triage nurse. Several AI-based solutions have been tested to assist triage nurses and standardize the triage process, having promising outcomes for the improvement of triage flow and patient outcomes [1]. For example, ML-based remote triage (ART) uses patient data and transfers it through a gateway to telemedicine servers within the hospital and is then utilized to triage patients into categories dependent on the emergency [14]. The digitalization of EDs and the capacity of current-generation computers allow for algorithm-based data evaluation and risk stratification for specific clinical endpoints beyond the triage level, providing a bright outlook on the implementation of AI in EDs [15].

Although AI provides many benefits, there is still a wide array of issues worth noting such as ethical issues relating to data sharing, FDA approvals, and addressing misconceptions about what AI is and does [6]. AI tools can also increase the risk of adverse outcomes in patients if the algorithms used are tuned to give overly confident results. It is thus important to promote education on the appropriate use and pitfalls of AI [16,12]. Current research has sought to analyze existing hospital quality assurance and quality improvement initiatives to create a template for designing clinical AI algorithms to reduce errors with AI integration into healthcare systems [17]. To enhance the standard of services provided, real-time medical interpretation can be used in conjunction with AI through the integration of large amounts of relevant health data into the healthcare system [18]. 

As mentioned above, studies exist that have reported the various ways AI and ML are being applied in specific components of ED triage, highlighting their potential to improve patient outcomes, reduce overcrowding, and/or streamline emergency care processes. This scoping review is the first to our knowledge to collate and summarize in a systematic way the major themes regarding the potential benefits of ML for triage in EDs, including 1) the possibility of enhanced decision-making by analyzing vast amounts of patient data quickly and accurately; 2) efficiency and speed may be increased in often crowded, fast-paced EDs by using AI-powered triage tools to streamline the process by quickly assessing patient symptoms, medical history, and other relevant factors; 3) ML algorithms can be trained on historical data to predict the severity of patients' conditions and identify those at risk of deteriorating, which can help ED staff proactively manage patient flow, allocate resources efficiently, and respond to emergencies more effectively; and 4) advanced data analytics can enable EDs to triage patients with a more comprehensive understanding of their health status. 

Objective

The purpose of this scoping review was to explore systematically and comprehensively the use and potential impacts of the use of AI and other forms of ML in the ED triaging process to determine the implications of its integration into the ED. The integration of AI and ML in healthcare overall has made considerable progress by providing its capability to extract insights from unexpected sources and drawing connections that humans may overlook [6,11,19,20]. AI’s efficacy in general healthcare raises the question of its impact on the ED triaging process. The use of AI in the ED has been shown to have positive outcomes in various criteria in current literature, but little is known about the use of AI in triaging patients in the ED. A scoping review on this interdisciplinary topic is justified given the expanse and inconsistency of the published literature and the time-sensitive nature of emergency care. The main review question was “How does using AI and other forms of ML in triage care affect clinical outcomes during ED visits?”

## Review

Methods

This scoping review was conducted following the Joanna Briggs Institute (JBI) methodology for scoping reviews [21].

Eligibility criteria

To meet inclusion criteria, articles had to be peer-reviewed, written in English, and based on primary data research studies that were published in U.S. journals between January 1, 2013, and December 31, 2023. Other criteria included 1) studies with patients needing to be admitted to hospital EDs, 2) AI must have been used when triaging a patient, and 3) patient outcomes must be represented. Theoretical articles, abstracts, opinion pieces, presentations, and non-governmental and governmental pieces were excluded. The review was also limited to studies published in the US to ensure relative standardization of the medical treatment being provided.

Information sources

**Figure 1:**
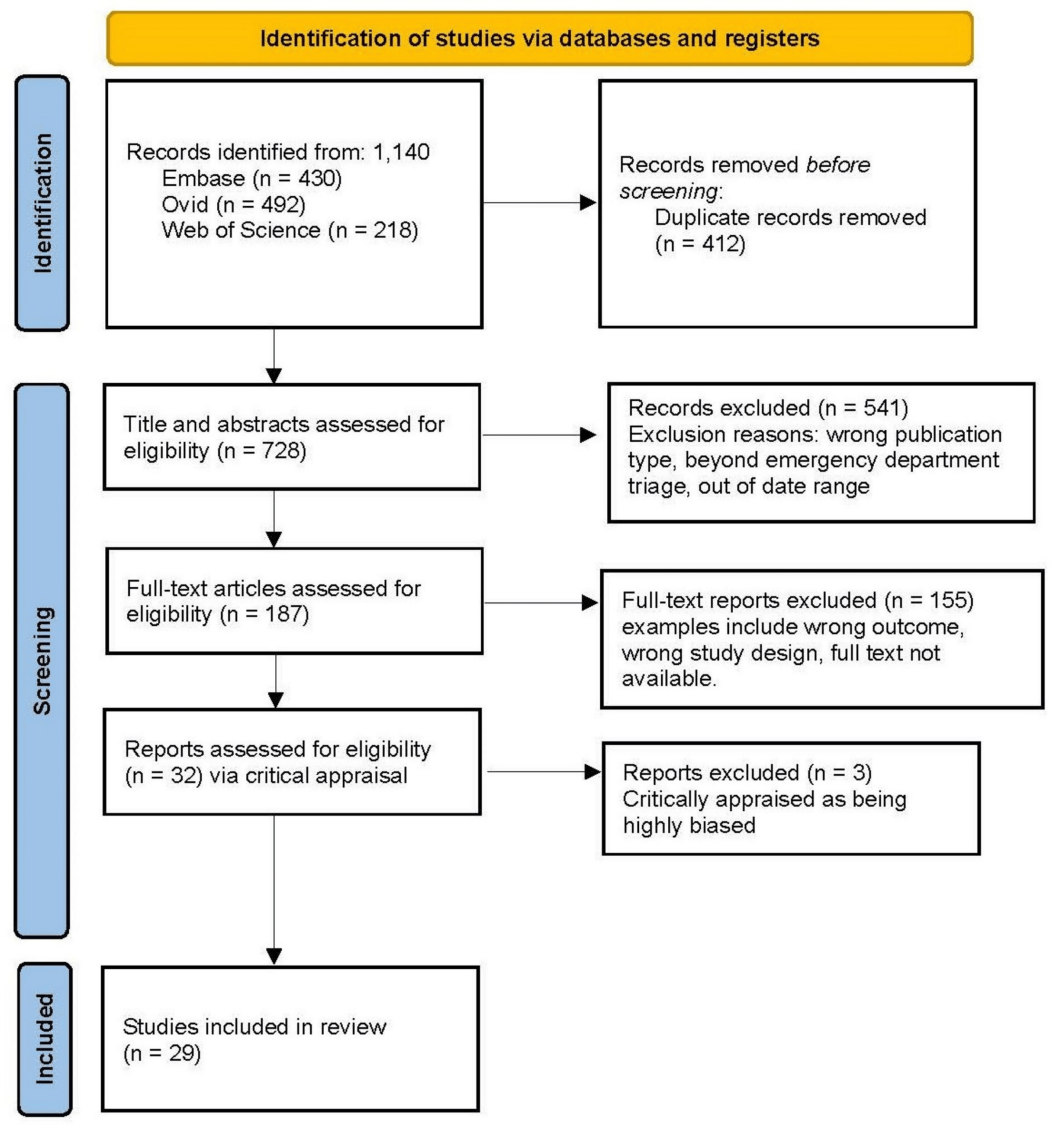
PRISMA flow diagram PRISMA: Preferred Reporting Items for Systematic Reviews and Meta-Analyses

The search initially identified a total of 1,140 citations. After removing 412 duplicates, 728 articles remained for further screening. Two authors screened each of the articles by title and abstract (with no disagreements between the screeners), resulting in 187 left for further full-text examination of their eligibility for inclusion by four other members of the research team, resulting in excluding 155 articles (did not meet the inclusion criteria); 74 articles (out of scope outcome); 55 articles (incorrect study design); 26 articles were not available as full-text). After two tiers of rigorous screening, 32 studies were selected for the final review. Any conflicts in publication selection were resolved by discussion with other team member reviewers. The PRISMA flowchart details the screening and selection process (Figure 1).

Search strategy

The research question was based on the Population, Concept, and Context (PCC) strategy, establishing P for patients admitted to EDs, C for capabilities of AI in effective patient triage, and C for hospital emergency rooms/departments. Based on these identifications, the review question was ‘How does using AI and other forms of ML in triage care affect clinical outcomes during ED visits?’ Data collection was conducted by searching the electronic databases EMBASE, Ovid MEDLINE, and Web of Science.

The search was conducted using controlled descriptors from the Medical Subject Headings (MeSH) using a search string that included the terms “artificial intelligence” OR “machine learning” AND “emergency ward” OR “emergency care” OR “emergency department” OR “emergency room” AND “patient triage” OR “triage” OR “triaging.” The descriptors were combined in different ways, aiming to broaden the searches. The terminological variations in the different languages, as well as synonyms, were used with the Boolean operators AND for the simultaneous occurrence of subjects and OR for the occurrence of their respective synonyms.

Selection of sources of evidence

All the authors discussed the results and inclusion criteria for consistency before the initial screening of the 232 articles generated in the primary search. Two authors then worked independently to evaluate the abstracts and titles of the publications to determine their relevance to our study. Thirty-two full-text articles appeared to be relevant for the final review. 

Critical appraisal of individual sources of evidence

The selection process places a strong emphasis on the quality and validity of articles, as they can potentially contain biases or skewed data that impact the overall integrity of the scoping review [21]. Consequently, a comprehensive evaluation of semifinalist articles occurred following the initial screening. This evaluation involved the use of the critical appraisal tools developed by the JBI, known for its reliability and ongoing improvement efforts. Most of the studies included were cohort studies with some inclusion of case-control, case series, and cross-sectional studies. The appropriate JBI checklist which considered research biases, overall coherence, and crucial sections that contribute to article quality was used. Two researchers independently conducted a detailed and blinded appraisal of the 32 articles chosen for the final review using the applicable JBI tools. Articles were then categorized into high, moderate, or low risk of bias based on their scores (below 50%, between 50% and 70%, and above 70%, respectively). Articles above 70% in the criteria were included while articles under 70% were considered at higher risk for bias and therefore excluded. Subsequently, the researchers engaged in a deliberative process to compare their appraisal scores. The relevance and quality of each article were thoroughly discussed, leading to a final consensus on selecting articles for inclusion in the review whereby three articles were excluded, resulting in 29 articles for the final review.

Data charting and extraction process

The data charting process was conducted using Microsoft Excel (Microsoft Corporation, Redmond, US). Team members extracted and charted data from each publication thought to be eligible for inclusion. Data were abstracted based on article characteristics (e.g., publication date, country of origin, publication language), algorithms utilized (e.g., decision tree, deep neural network, gradient boosting), the respective area under the curve (AUC) for the algorithm utilized, and the impact of AI on ED triage (e.g., decreasing mortality, predicting duration of hospital stay, assisting in clinical decision-making). Conflicts in publication selection were resolved by discussion with other team members.

Results

The studies included were retrospective, included observations of patients admitted to the ED, and addressed the use of AI for triage. All articles were published in the US between 2018 and 2023.

Triage efficiency

The collective findings from several studies in this scoping review discussed the impact of AI applications on ED triage efficiency [22-24]. Gao et al.’s study found that implementing an accurate triage model can alleviate the workload of medical staff, enhance emergency treatment efficiency, and optimize the allocation of medical resources [22]. The authors highlighted the limitations of current triage systems and emphasized the importance of an accurate and efficient triage model for ensuring prompt assessment and treatment of emergency patients. Their extreme gradient boosting (XGBoost) ML algorithm achieved a sensitivity of 83.6%, a specificity of 78.9%, and overall prediction accuracy of 80.2% in predicting triage preparation [22]. Another study reported the development of a low-dimensional ML model to predict whether a patient would be admitted or discharged from the pediatric ED, resulting in an area under the receiver operating characteristic curve (AUROC) of 0.853 [24]. They concluded that early prediction of admission and discharge probabilities during the ED triage process could be utilized to enhance patient flow and contribute to more effective bed management. AI models were found to have the potential to streamline ED operations, mitigate challenges associated with ED crowding, as well as contribute to the efficient management of ED resources [23].

Six of the 29 studies investigated ML models that consistently demonstrated superior discrimination abilities compared to conventional triage systems [25-30]. In one report, these models exhibited reduced under-triaging and over-triaging in traditional triage levels while displaying heightened sensitivity in identifying critically ill patients [25]. Additionally, the implementation of ML systems was associated with a significant reduction in the rate of mistriage of ED patients who were critically ill and reduced the mistriage rate to 0.9% compared to using a traditional triage system (1.2%) [29]. The results from Raita et al.’s study that used a large dataset of adult ED visits revealed that four ML models outperformed the Emergency Severity Index (ESI) in forecasting outcomes of critical care and hospitalization, with higher discriminatory abilities and reduced under-triaged patients in levels three to five of ESI triage [30]. These studies underscored the potential of ML in enhancing the accuracy of triage decisions to improve patient outcomes in emergency settings.

Some reports reinforced the superiority of developed ML models over conventional triage systems [27,28,31]. One such study demonstrated an AUROC of 0.991 for predicting critical outcomes in pediatric ED visitors, whereas another study demonstrated the superiority of deep learning models in accurately identifying critically ill patients, surpassing the performance of the ESI and vital sign triggers [27,28]. ML algorithm Random Forest demonstrated superior accuracy, precision, and specificity compared to other ML models in distinguishing high and low case severity, predicting triage levels, as well as comparing the distribution of patients across triage levels with the existing expert hospital triage system [31].

Another study reported integrating data from 166,175 encounters with ED patients and found that the triage model KATE™ demonstrated a 75.7% rate of accuracy in predicting ESI acuity assignments [32]. In comparison, triage nurses had an accuracy of 59.8%, and individual study clinicians achieved 75.3%. These findings revealed that KATE™’s accuracy surpassed that of an average triage nurse by 26.9% and showed a 93.2% improvement on the boundary between assignments in triage levels 2 and 3 of ESI.

Xiao et al.’s study aimed to enhance ED triage with two ML-based methods, TransNet and TextRNN. TextRNN achieved a success rate of 86.23% in predicting severity levels and 94.30% for clinical departments across 161,198 ED visits [33]. Additionally, TransNet exhibited higher sensitivities, with rates of 84.08% for severity levels and 90.05% for clinical departments, along with specificities of 76.48% and 95.16%, respectively.

Predictive modeling for disease identification

Numerous articles in this review aimed to leverage predictive modeling for disease identification and risk assessment within the scope of ED triage and care [26,34-37]. ML algorithms have been employed in the early identification and prediction of critical conditions, including bacteremia and cardiopulmonary arrest [35,36]. As showcased by Chen et al.’s study, ML algorithms demonstrated their capability to provide more accurate and timely predictions for critical care patients compared to emergency physicians, enabling earlier identification and intervention [34]. This investigation involved the development of an ML model that outperformed other models, demonstrating an improved sensitivity and accuracy in critical outcome prediction compared to the ED physicians using data from Taiwan’s Taipei Medical University-Shuang Ho Hospital. Specifically, the model yielded a sensitivity of 0.95 and an accuracy of 0.90 in comparison to that of the emergency physicians who achieved 0.41 and 0.67, respectively [34]. Other studies also highlighted the effectiveness of ML models in the prediction of TBI case mortality, citing average accuracy rates ranging from 93.2% to 95.4% [26,37]. The logistic regression (LR)-based model, an ML algorithm, was identified as the top-performing model for mortality risk prediction in TBI patients, achieving a high AUROC of 0.925 [37].

Researchers from Chang et al.’s study utilized an XGBoost algorithm to build six distinct and independent models designed for each critical intervention, achieving high specificity and sensitivity using only triage data available early in the ED stay [38]. The study’s model exhibited high accuracy, with AUROC values ranging from 0.909 to 0.962 for predicting the necessity for the six specific interventions: intubation, oxygen therapy, arterial line insertion, high-flow nasal cannula, massive transfusion protocol, and inotropes or vasopressors administration.

Three studies identified a demand for decision-support tools to aid in the identification of certain conditions and triage of ED patients, including those diagnosed with Crohn's disease, sepsis, and in-hospital cardiac arrest [39-41]. ML models pinpointed clinical complications, such as intra-abdominal abscesses presented in hospitalized patients with Crohn’s disease. Moreover, these investigations further revealed that factors like CRP, hemoglobin, WBC, age, biological therapy, and BUN were associative contributors to intra-abdominal abscesses. Concerning sepsis patients, the XGBoost algorithm was validated to stratify ED patients with significantly better discrimination when compared to current clinical sepsis risk scores, such as systemic inflammatory response syndrome (SIRS) and qSOFA [40]. Furthermore, ML was also shown to predict in-hospital cardiac arrest in the ED based on triage data, with the ML models outperforming the National Early Warning Score 2 [41].

The integration of AI into the triage process yielded significant enhancements in predictive accuracy and risk assessment. One such study that supported this notion used AI models, including LR and XGBoost, that exhibited notable improvements when incorporating the chief complaint as a predictor [36]. The LR model showed a 2% increase in the AUROC and an 8% increase in average precision (AP). Similarly, the XGBoost model experienced a 4% increase in AP with no change in AUROC.

Predicting hospital admission

Choi et al.’s study addressed the issue of unnecessary blood cultures and the delayed treatment of bacteremia in the ED [35]. The authors created and validated ML models to anticipate bacteremia at the ED during the stages of triage and disposition. The study found that the admission rate in the validation dataset would have risen by 3.3% if all patients identified as high risk by the XGB model had been admitted; however, the proportion of discharged patients with bacteremia would have decreased by 36.2%. Another study demonstrated the potential of ML techniques in forecasting the admission of ED patients based on conventional biomarkers and coagulation tests with F-measure and AUROC values ranging from 0.679 to 0.708 and 0.734 to 0.774, respectively [42]. Goto et al.'s study predicting hospitalization outcomes of patients with asthma or COPD exacerbation revealed that the traditional LR with ESI had a C-statistic of 0.64, while the ML models had a greater discriminative ability, with C-statistics ranging from 0.82 to 0.83 [43]. The random forest model yielded the greatest ability, with a C-statistic of 0.83. Similarly, another study found that ML models demonstrated superior performance in hospitalization outcome predictability compared to the traditional ESI approach [30].

One study focused on forecasting hospital admission during ED triage using ML techniques considering both patient history and triage information [44]. It was found that models incorporating historical data encompassing prior healthcare utilization, medical history, past laboratory results and vital signs, prior imaging records, outpatient medications, and detailed demographic information in addition to triage information alone yielded the highest AUROC values (>0.90) for predicting hospital admission [44]. Another study achieved a high accuracy in predicting hospitalizations for pediatric ED visitors using ML models that outperformed traditional pediatric triage systems including one model with an AUROC of 0.943 for hospitalizations [27]. Key findings from a study predicting the necessity of neurosciences intensive care unit (NSICU) admission 30 minutes after arriving at the ED include an AUROC of 0.93 using both structured and free text data [45]. When examined separately, a model trained exclusively on text data yielded an AUROC of 0.90, and a model using only structured data achieved an AUROC of 0.92. The combined model demonstrated a sensitivity of 58% in detecting NSICU admission with a false positive rate of 1:100 (99% specificity). Another study conducted using data from a tertiary teaching hospital in Taiwan focused on urgent level-3 patients and found that using an AI model achieved an AUROC of 0.8004 and accurately determined the necessity of hospitalization for patients requiring urgent attention [23].

ML models also used triage data to predict the need for hospitalization of ED patients with fall-related fractures [46]. The corresponding study found that neural network models were the top performers in predictive accuracy. Their results also revealed that older patients, females, patients arriving by ambulance, and those with certain chief complaints were more likely to be hospitalized. Another study in this review analyzed data from 29,354 pediatric patients with asthma exacerbation seen at two pediatric EDs over four years and found that the gradient boosting machine's model achieved the highest success in forecasting hospitalization need, with an AUROC of 0.85 [47].

Improving resource allocation and quality of patient care

Findings from a study looking at ED patients presenting with syncope found that predicting the length of stay can improve patient care by aiding hospitals in allocating resources more efficiently and ensuring that patients receive the appropriate level of care [48]. The results indicated that the ML algorithm could accurately predict a longer length of stay to assist in planning for necessary interventions and care management, ultimately leading to shorter wait times and reduced risk of extended hospital stays [48].

The authors of one article introduced the VitalML framework, a multimodal ML approach, to assess which patients would develop hypoxia, hypotension, or tachycardia within the following 90 minutes [49]. They found that the ML model accurately predicted the onset of clinical decompensation in initially stable patients in the ED using continuous physiologic data from the first 15 minutes of monitoring.

Wu et al. discovered that the LASSO regression model is an accurate and promising prediction tool for identifying critically ill patients presenting with chest pain to the ED [50]. The authors concluded that the model could assist hospitals in efficiently allocating sparse resources while preserving the safety of chest pain patients at high risk.

A summary of the 29 articles included in this review is displayed alphabetically by author in Table 1. 

**Table 1:** Summary table of the articles included in the review AUC, area under the ROC curve (ROC is a probability curve and AUC represents the degree or measure of separability); AUPRC, area under the PRC Curve, DNN, deep neural networks; ED, emergency department; ESI, emergency severity index; GB, gradient boosting; GBM, gradient boosting machine; GRACE, The Global Registry of Acute Coronary Events (to identify patients in the coronary care unit or ED at the greatest risk of adverse events after acute coronary syndrome); HEART, history, electrocardiogram, age, risk factors, and troponin; ICU, intensive care unit; IHCA, in-hospital cardiac arrest; KATE™, Knowledge Assessment Teaching Engine (AI providing 24/7 real-time clinical risk guidance for EDs); KNN, k-nearest neighbors (algorithm); KTAS, Korean triage and acuity scale; LASSO, Least Absolute Shrinkage and Selection Operator; LightGBM, gradient boosting framework that uses tree-based learning algorithms; LR, logistic regression; ML, machine learning; MLP, multi-layer perceptron (algorithm); MTS, Manchester Triage System; NB, Naïve Bayes (algorithm); NHAMCS, The National Hospital Ambulatory Medical Care Survey; NPV, negative predictive value; PPV, positive predictive value; qSOFA, quick SOFA (score for Sepsis identified high-risk patients for in-hospital mortality; suspected infection outside ICU); REP, reduced error pruning; RF, random forest (algorithm); SIRS, systemic inflammatory response syndrome; SVM, support vector machine (algorithm); TextCNN, the convolutional neural network for text (deep learning algorithm); TIMI, thrombolysis in myocardial infarction; XGBoost, extreme gradient boosting (machine learning algorithm that belongs to the ensemble learning category, specifically the gradient boosting framework); AI, artificial intelligence; ANN, artificial neuronal network; DOA, dead on arrival; NHAMCS, National Hospital and Ambulatory Medical Care Survey; NEMC, National Economic Advisory Council; NEDS, National Emergency Department

Author	Purpose	Study design	Sample	Quantitative results (AUC unless otherwise specified)	ED effect	Limitations
Chang, 2022 [38]	ML to predict critical interventions in severe patients presenting to ED triage using vital signs and KTAS	Retrospective observational	137,883 adults nonDOA in Korea	XGBoost algorithm. LR AUC - A-line insertion 0.913, oxygen therapy: 0.909, high-flow nasal cannula: 0.962, intubation: 0.945, massive transfusion protocol: 0.920, and inotropes: 0.899	A high degree of accuracy and decreased time needed to provide services to critical patients.	Predicting intervention, not triage accuracy. Single centers, which could have selection bias, did not consider patients who receive palliative care over intensive care.
Chen, 2023 [34]	Developed a machine-learning-based prediction model and evaluated its performance against other methods and 13 ED physicians, which displayed improved identification of patients with critical outcomes (IHCA or ICU admission).	Retrospective cohort	171,275 adults in Taiwan	LR (0.828), RF (0.789), DNN (0.874), gradient boost (0.783), Naive Bayes (0.785), multilayer perceptron (0.765), TextCNN (0.786), Bert (0.810), BiLSTM (0.807), BiLSTM+TR (0.844), final model: 0.874	The generated prediction model outperforms other prediction models and has better sensitivity and accuracy compared to emergency physicians.	Pre-processing large free text data, large amounts of missing data, and data from one hospital.
Choi, 2022 [35]	Develop ML models to predict bacteremia at ED triage and disposition stages.	Retrospective cohort	24,768 adults with two blood tests; Korea	LR (0.728), RF (0.782), XGB (0.747)	The triage XGB model could be used to identify patients with a low risk of bacteremia immediately after initial ED triage. The disposition XGB model showed excellent discriminative performance and was superior to other ML models, qSOFA, and SIRS. This can facilitate early ED disposition decisions.	No exact hospital name where data was taken. Data from a single ED limits generalizability. Low-risk patients might not have had blood samples taken and therefore were not included. No data on the volume of blood taken. Cutoff probability thresholds for the risk of bacteremia weren’t chosen based on data regarding hospital outcomes.
Feretzakis, 2022 [42]	Eight ML algorithms generated models that can reliably predict the hospital admission of patients seen in the ED based on common laboratory tests and basic demographics.	Retrospective observational	3,204 in Greece	LR: 0.765, RF: 0.789, DNN: 0.74, LR with ada boost: 0.731, LR with logit boost: 0.757	The utilization of AI may have a favorable impact on the future of emergency medicine. Investigate if a model that is easy to access with a low cost can identify hospital admissions without considering clinical data.	Did not include clinical parameters such as vital signs and ESI to keep low cost. Missing lab values.
Fernandes, 2020 [36]	Predict risk of mortality and cardiopulmonary arrest in ED presentations using ML and natural language processing	Retrospective cohort	235,826 adults in Portugal	LR: 0.95, RF: 0.94, GB: 0.96	The XGBoost model could identify patients with a higher risk of a composite outcome in MTS-3 and presented a lower number of false negative classifications for MTS-1 and MTS-2. While the reference model presented higher recall for MTS-1 and MTS-2, the XGBoost model proved to have a higher sensitivity in identifying patients assigned to MTS-3. Complement the already existing triage system with an ML model and avoid under-triaging.	Only includes MTS 1-3, excludes nonurgent. The model doesn’t consider other complications.
Gao, 2022 [22]	Developing and analyzing an ED triage model using ML algorithms with medical big data	Retrospective	276,164 aged 14 and up in Beijing	XGB prediction accuracy rate: 0.8257, level 1 - 0.9629, level 2 - 0.9554, level 3 - 0.912, level 4 - 0.9296	Data that reduce the number of triage nurses at emergency triage stations and, to some extent, improves their working efficiency.	Data from a single institution, subjectivity. Triage standards provided at different times, in diverse scenarios, by various triage staff, and by the same triage staff with different physical and mental states may not be the same, resulting in poor stability in the triage results.
Goto, 2018 [43]	ML approaches to predict disposition of asthma and COPD exacerbations in the ED	Retrospective cohort	3,206 patients, mean age 52, presenting to ED with obstructive airway disease exacerbation, taken from NHAMCS, US	Critical care outcome C-statistics: reference model (0.68), LR with LASSO (0.79), RF (0.76), GB decision tree (0.8), DNN (0.79). Hospitalization outcome C-statistics: Reference model (0.64), LR with LASSO (0.82), RF (0.83), GB decision tree (0.82), DNN (0.82)	The use of ML significantly improved the ability to predict two clinical outcomes (critical care and hospitalization) over the traditional approach using ESI information.	Patients with missing information were excluded which could create selection bias. Survey data may have misclassification and NHAMCS does not measure some clinical variables.
Goto, 2019 [25]	Predicting critical care and hospitalization outcomes using a reference model and four ML models in children presenting to the ED	Retrospective cohort	52,037 children from NHAMCS data, US	Critical care outcome C-statistics: reference model (0.78), LR with LASSO (0.84), RF (0.85), GB decision tree (0.84), DNN (0.85). Hospitalization outcome C-statistics: Reference model (0.73), LR with LASSO (0.78), RF (0.80), GB decision tree (0.80), DNN (0.80)	ML in ED triage improved the discriminative ability to predict clinical outcomes compared with conventional triage, as well as had a high sensitivity for predicting critical care outcomes. This would reduce the number of under-triaged children in triage levels 3-5 and avoid over-triaging children who are less ill.	Excluded visits with no information on conventional triage classification. Thresholds for outcomes may be different between EDs. All limitations listed had proactive planned solutions by the researchers.
Hong, 2018 [44]	Predicting hospital admission at ED triage using ML models	Retrospective	202,953 adults from 3 EDs, Yale New Haven Health System, US	Only triage: LR (0.865), XGBoost (0.874), DNN (0.873). Only history: LR (0.862), XGBoost (0.871), DNN (0.872). Full: LR (0.909), XGBoost (0.924), DNN (0.920). Top variables: XGBoost (0.910)	ML can predict hospital admission at ED triage, especially with the addition of patient history and other variables. These features can be used to create a model that can be implemented into EHR systems as clinical decision support.	Only used patient history gathered from previous ED visits. Data came from a hospital system that includes multiple EDs. Cannot address the appropriateness of ED providers' prior decisions. Doesn’t address implementation and efficacy barriers in clinical practice.
Hsu, 2021 [26]	Create a model that predicts in-hospital mortality in TBI patients presenting to the ED based on clinical measures and demographics	Retrospective cohort	3,331 age 16+ with TBI, Taiwan	J48 (0.82), RF (0.921), random tree (0.735), REP tree (0.846), KNN (0.716), SVM (0.71), NB (0.917)	These algorithms provide evidence from ML to aid in clinical decision-making, increase provider awareness of clinical prognosis in TBI patients, and possibly prevent death.	Did not take into account underlying medical conditions, comorbidities, type of treatment received in the ED or hospital, imaging, or findings. Data from a single site and study was limited by the small number of patients who died.
Hwang, 2022 [27]	ML to predict critical cases and hospitalization among children visiting the ED	Retrospective	262,1710 under 15 years of age from NEMC, Korea	RF AUROC 0.991 (critical cases) and 0.943 (hospitalizations). Conventional predication AUROC 0.844 (critical cases), 0.680 (hospitalizations)	ML models using a nationwide database can predict critical hospitalizations of pediatric ED visits more effectively than the conventional triage method.	Although the model showed high AUROC values, the AUPRC of the entire dataset was low (0.640 for critical cases and 0.729 for hospitalization), which was probably due to the imbalanced dataset.
Ivanov, 2021 [32]	Determine if EHR data can be extracted with clinical natural language processing and KATE™, an ML algorithm, to produce more accurate ESI predictive models	Retrospective	Randomly selected 800 from 147,052 encounters, used 729, US	Across the study sites: KATE™’s accuracy (75.7%), average nurse accuracy 59.8%, P < .001 site a kate accuracy was higher than the average nurse b combined for study sites not significantly different that of each clinicians. demonstrated esi triage acuity boundary	KATE™, using the XGBoost gradient, demonstrated significantly higher ESI accuracy, measured against the consensus of the expert clinicians supported by the ESI Handbook, than the nurses for all gold records from each study site and for each triage acuity level.	Limitation: when comparing to nurse results, could vary depending on nurse experience, age, etc.
Joseph, 2020 [28]	Deep-learning approaches to identify critically ill patients at ED triage using limited information	Retrospective cross-sectional	445,925 adult patients, Northeastern US	XGBoost (0.82), neural network structured data (0.812), neural network combined data (0.857), LR (0.805). The final neural network model classified critically ill patients with AUC 0.851 (95% CI = 0.849–0.852), reflecting a total sensitivity of 0.845 (95% CI = 0.844–0.846)	Deep‐learning approaches to identifying critically ill patients at ED triage, neural network, and gradient‐boosting models demonstrated significantly higher accuracy than traditional methods of triage, suggesting that these models have the potential to significantly enhance the triage process.	Data from a single center with a higher proportion of critically ill patients than other studies. ICU admission criteria and availability can vary across hospitals. Abnormal vital signs can enhance the accuracy of any predictive test for ICU admission. Some critically ill patients who respond to ED treatment may be mislabeled as not critically ill.
Klang, 2021 [45]	ML model based on tabular-free text data to predict neuroscience intensive care unit admission	Retrospective cohort	412,858 adults, Mount Sinai Hospital, an academic medical center in New York City, NY, US	Text and tabular data combined with XGBoost, final AUC 0.93	Such a model could be used by neurocritical care experts and clinical stakeholders, such as ED clinicians and nursing managers, to identify patients who might need an NSICU bed early in the ED triage process.	Data from a single center, incorporated potentially non-generalizable factors like resource availability, triage procedures, practice styles, etc. that may vary across institutions. Did not compare the performance of the model to trained physicians.
Lee, J. 2021 [23]	Predicting hospitalization for urgent patients in the ED using AI	Retrospective cross-sectional	282,971 adults, Taiwan	DNN validation AUC using a few triage metrics (0.8004). Best performance predicting nontraumatic adults (0.8166).	This model can increase ED physician confidence in their decisions regarding patient disposition, and allow for quicker initiation of hospital admission.	The single medical center did not include oxygen saturation and did not compare the model with other computer or human (nurse or physician) models.
Lee, S. 2023 [48]	Using ANN ML to predict the length of stay and mortality for patients admitted from the ED with syncope	Retrospective cohort	4,645,483 presenting to ED with syncope, NEDS data, US	ANN 0.78 <0 days, 0.79 <24 hrs., 0.81 <48 hrs., 0.84 <4 days, 0.88 <7 days. Able to predict short or <48 hrs length of stay in syncope patients with an AUC of 0.81, compared to long (>48 hrs) length of stay	NNS showed promise in predicting length of stay for patients presenting with syncope with a fair to good performance in AUC ranging from the same day discharge, short to long length of stay. In order to perform clinically, the AUC must be 0.90 or higher, thus there is much improvement needed to be made to the ML algorithm.	Limited by the information available on the NEDS dataset, assumes coding and diagnoses are accurate, limited to a single encounter, unknown comorbidity diagnoses made at an initial encounter or during hospitalization.
Leonard, 2022 [24]	Development of a low-dimensional model to predict admissions from triage at a pediatric ED	Retrospective	72,229: 48,861 used for model development, 3,447 to test the model. <18 years old, Ireland ED	GBM model (33 variables), highest AUC ( 0.853), making it the best-performing model based on AUC alone. A low-dimensional GBMl model was made with the top 8 variables, AUC (0.835). Naive Bayes (0.812), LR (0.845)	Similar capabilities of a model based on 8 variables as one based on 33. Models that predict admission and discharge can be used for additional decision support information, and allow nurses to request beds while waiting for clinical decision-making or fast track for discharge. Help patient flow and hospital overcrowding.	One ED in Ireland. Possible limitations from variable presenting complaints and triage categories.
Levartovsky, 2021 [39]	Using ML to predict the presence of intra-abdominal abscesses in Crohn's patients visiting the ED	Retrospective cohort	309 aged 16 years and older with Crohn's disease, Sheba Medical Center, Israel	LR (0.816), RF (0.817)	An ML decision support system can be implemented into EHR to guide physician clinical decisions regarding imaging, discharging, and admission.	Only included patients who underwent abdominal imaging, therefore already have a high clinical suspicion of an intra-abdominal abscess. Excluded patients who were discharged from the ED assuming they had uncomplicated disease.
Lin, 2021 [40]	Developing an ML model to identify sepsis in the ED	Retrospective cohort	8,296 adults, Taiwan	Gradient boosting (0.86), XGBoost (PPV: 0.47, NPV: 0.94) were higher than that of SIRS (PPV: 0.34, NPV: 0.77) and qSOFA (PPV: 0.76, NPV: 0.79)	XGBoost model outperformed the pre-existing conventional tools in identifying sepsis patients (qSOFA and SIRS). ML can improve sepsis outcomes by early identification and facilitating timely sepsis care.	Differences in patient characteristics are key predictors but can reduce model performance. Heterogeneity in study designs limits the comparison of performance in this model to the previous.
Liu, 2021 [13]	Development and validation of a practical machine-learning triage algorithm for the detection of patients in need of critical care in the ED	Retrospective data collection, prospective cohort testing design	22,272 adults, Singapore	LR (0.843) MLS catboost (0.875)	Mode of arrival was the most important triage feature. Age can be viewed as a continuous variable rather than a blunt cut-off. Pulse pressure and shock index were found to be beneficial more than SBP.	The model used some features like arrival time, arrival mode, and initial triage level, which can vary based on areas or facilities.
Lu, 2022 [41]	ML to predict in-hospital cardiac arrest from patients presenting in the ED	Retrospective cohort	316,465 adults, Taiwan	LR (0.905), RF (0.931), decision tree (0.915), gradient boosting (0.93)	ML models can successfully predict ED cardiac arrest based solely on the clinical features that are readily available at ED triage and therefore can aid identification of high-risk patients and prevent deaths.	3,252 records were excluded due to missing data. Data from only one hospital. ML had high specificity but modest sensitivity.
Pai, 2022 [46]	Predicting hospital admission from ED triage data for patients presenting with fall-related fractures	Retrospective cohort	6,335 patients presenting with a fall to the ED 2013-2016 in Pennsylvania, US hospital	LR (0.936), DNN (0.983), discriminant analysis (0.93), kNN (0.936)	ML can shorten the time to admit and decrease overall ED wait time by making efficient movement of patients out of the ED. The admission process can be streamlined and give priority to those more likely to be admitted. The inpatient department can be notified earlier about admission so allocation of staff and beds can be planned effectively. DNN performed best, but LR models are likely easier to integrate with EHR interfaces that hospitals use.	The number of fall-related ED visits might be underestimated. The study is limited to one hospital. Possible data recording errors despite effort assigning an ICD-10 code to each patient diagnosis.
Patel, 2018 [47]	Developing four ML approaches using triage data to predict the risk of hospitalization for an acute exacerbation of pediatric asthma	Retrospective cohort	29354 pediatric asthma (age 2-18 years)	LR (0.82), RF (0.82), decision tree (0.68), GB (0.85)	ML models can be used in triage to differentiate low and high-risk patients to improve efficiency.	Data from one institution. Did not evaluate the necessity of hospitalization or consider progression of illness or non-clinical reasons for hospitalization.
Raita, 2019 [30]	ED triage prediction of clinical outcomes using ML models	Retrospective cohort	135,470 adults, NHAMCS survey data	Critical care: LR (0.84), RF(0.85), DNN (0.86), GB (0.85). Hospitalization: LR (0.81), RF (0.81), DNN (0.82), GB (0.82)	The current study corroborates the promise suggested by these recent studies and extends them by demonstrating the superior predictive abilities of modern ML models over the conventional model in a large population of adults in the ED. ML can also reduce the under-triaging of critically ill individuals.	Excluded samples with missing information. ICU and admission thresholds depend on location and can vary between ED physicians.
Sundrani, 2023 [49]	A multimodal learning framework for predicting patient decompensation from continuous physiologic monitoring in the ED	Retrospective cohort	19,847 adult ED visits	Predicted tachycardia (0.836), hypotension (0.802), hypoxia (0.802)	For each outcome, the models that used features from 15 minutes of passive monitoring significantly outperformed models restricted to conventional triage features. For some outcomes and prediction windows, engineered and learned waveform features improve discrimination over vital sign trends alone. This approach could be used to improve the triage of initially stable patients at risk for decompensation and could be applied continuously for real-time estimates of near-term clinical deterioration.	Data taken from a single academic center may not be generalized to a different setting. The outcome of interest represents a small portion of all ED visits.
Tu, 2022 [37]	A computer-assisted system for early mortality risk prediction in patients with TBI using AI algorithms in emergency room triage	Retrospective Observational	18,249 adult TBI injury patients in the Chi Mei Medical Group	LR(0.925), XGboost(0.871), random forest(0.87), MLP, LightGBM	Without clinical laboratory data and imaging studies, the results showed that the LR algorithm was the best algorithm to predict the mortality risk in patients with TBI in the emergency room triage setting. The system can easily obtain the 12 predicted variables during initial triage which can provide earlier mortality prediction.	Feature variables could have been miscoded or biased and affect the mortality of TBI patients and there was no evaluation of variable features (coagulopathy, brain CT scans, surgery, and complications). Difficult to generalize data to other hospitals and countries due to country-specific measurements.
Wolff, 2019 [31]	Provide a methodological proposal for pediatric triage ML model construction based on clinical outcomes	Retrospective Cohort	189,718 patients	Hospitalization: LR (0.8), DNN (0.77), NB (0.78). Death: LR (0.86), DNN (0.78), NB (0.77)	From the model viewpoint, their results showed successful experimentation of different ML techniques that have a recent interest in scientific research. There is great potential for them to be used in real clinical settings as a triage decision support tool.	Data is taken from a single institution, and records determined to be "incomplete or erroneous" were excluded.
Wu, 2021 [50]	ML LASSO regression model to predict critical care outcomes in patients with chest pain visiting the ED	Retrospective case-control	3,146 patients with acute non-traumatic chest pain in the ED	LASSO regression model (0.953)	Compared to well-known clinical risk scores, HEART, GRACE, and TIMI scores, their LASSO regression model had a superior performance in predicting critical care outcomes in patients with chest pain. Moreover, the model minimized the potential over-predicted and under-predicted critical care outcomes that could result in excessive resource allocation to low-risk patients and insufficient treatment of high-risk patients.	Single-center studies at a tertiary provincial ED in China can be limited by institutional factors and potential selection bias. Intrinsic limitation of case-control studies of medical history data. External validation is required before routine clinical use. Other clinical features were not studied such as heart rate variability. Indication and clinical threshold of ICU admissions vary.
Xiao, 2023 [33]	Criticality and clinical department prediction of ED patients using ML based on heterogeneous medical data	Retrospective	308,834 ED patient visits from Peking University People's Hospital, China	TransNet, TextRNN AUC (0.93, 0.98), accuracy (0.86, 0.94), sensitivity (0.85, 0.89), specificity (0.79, 0.94)	These findings suggest that the proposed TextRNN and TransNet models significantly reduce the under-triage and over-triage issues compared to manual triage. the proposed models, TransNet and TextRNN, outperform reference models and can accurately predict patient severity level and clinical department.	Recognition accuracy for severity levels and clinical departments was low. Quantification of the efficiency issue was not possible because it was difficult to quantify the allocation of resources. The proposed models only retrospectively provided data, not real-time ED triage.

Table 2 displays a synopsis of the main effects of AI and ML in ED triage for each article in the review. 

**Table 2:** The main effects of AI and ML in ED triage AI, artificial intelligence; ML, machine learning; ED, emergency department; ESI, emergency severity index; LASSO, Least Absolute Shrinkage and Selection Operator; AUC, area under the ROC curve; XGBoost, extreme gradient boosting; LR, logistic regression

Author	The main effects of AI and ML in Ed triage
Chang, 2022 [38]	A high degree of accuracy and decreased time needed to provide services to critical patients.
Chen, 2023 [34]	The generated prediction model outperforms other prediction models and has better sensitivity and accuracy compared to emergency physicians.
Choi, 2022 [35]	The triage model used in the study could be used to identify patients with a low risk of bacteremia immediately after initial emergency room triage and showed excellent discriminative performance in facilitating early emergency room disposition decisions.
Feretzakis, 2022 [42]	The utilization of AI may have a favorable impact on the future of emergency medicine.
Fernandes, 2020 [36]	The XGBoost model proved to have a higher sensitivity in identifying patients assigned to the Manchester Triage System-3 and suggested complementing the already existing triage system with a ML model and avoiding under-triaging.
Gao, 2022 [22]	AI reduced the number of triage nurses at emergency triage stations and to some extent improved their working efficiency.
Goto, 2018 [43]	The use of ML significantly improved the ability to predict two clinical outcomes (critical care and hospitalization) over the traditional approach using ESI information.
Goto, 2019 [25]	ML in emergency room triage improved the discriminative ability to predict clinical outcomes compared with conventional triage and had a high sensitivity for predicting critical care outcomes to reduce the number of under-triaged children in higher triage levels and avoid over-triaging children who are less ill.
Hong, 2018 [44]	ML can predict hospital admission at emergency room triage. These features can be used to create a model that can be implemented into electronic health records systems as clinical decision support.
Hsu, 2021 [26]	These algorithms provided evidence from ML to aid in clinical decision-making, increase provider awareness of clinical prognosis, and possibly prevent death.
Hwang, 2022 [27]	ML models using a nationwide database can predict critical hospitalizations of pediatric ED visits more effectively than the conventional triage method.
Ivanov, 2021 [32]	ML models demonstrated significantly higher ESI accuracy, measured against the consensus of the expert clinicians supported by the ESI Handbook, than the nurses for all gold records from each study site and for each triage acuity level.
Joseph, 2020 [28]	Deep learning approaches to identifying critically ill patients at ED triage, neural network, and gradient‐boosting models demonstrated significantly higher accuracy than traditional methods of triage, suggesting that these models have the potential to significantly enhance the triage process.
Klang, 2021 [45]	A deep learning model could be used by neurocritical care experts and clinical stakeholders, such as ED clinicians and nursing managers, to identify patients who might need a neuroscience intensive care unit bed early in the ED triage process.
Lee, J. 2021 [23]	This model can increase ED physicians’ confidence in their decisions regarding patient disposition and allow for quicker initiation of hospital admission.
Lee, S. 2023 [48]	Showed promise in predicting length of stay for patients presenting with syncope with a fair to good performance in the AUC ranging from the same day discharge to long length of stay.
Leonard, 2022 [24]	Models that predict admission and discharge can be used for additional decision support information, allow nurses to request beds while waiting for clinical decision-making, or fast track for discharge. Also helps patient flow and hospital overcrowding.
Levartovsky, 2021 [39]	A ML decision support system can be implemented into electronic health records to guide physician clinical decisions regarding imaging, discharging, and admission.
Lin, 2021 [40]	The ML model outperformed the pre-existing conventional tools in identifying sepsis patients.
Liu, 2021 [13]	In the model, the mode of arrival was the most important triage feature; pulse pressure and shock index were found to be beneficial.
Lu, 2022 [41]	ML models can successfully predict ED cardiac arrest based on the clinical features available at triage, aiding the identification of high-risk patients and preventing deaths.
Pai, 2022 [46]	ML can shorten admission time and decrease overall ED wait time by making efficient movement of patients out of the ED.
Patel, 2018 [47]	ML models can be used in triage to differentiate low- and high-risk patients to improve efficiency.
Raita, 2019 [30]	Demonstrates the superior predictive abilities of modern ML models over the conventional model in a large population of adults in the emergency room and reduces under-triaging of critically ill patients.
Sundrani, 2023 [49]	The models that used features from 15 minutes of passive monitoring significantly outperformed models restricted to conventional triage features. This approach could be used to improve the triage of initially stable patients at risk for decompensation and could be applied continuously for real-time estimates of near-term clinical deterioration.
Tu, 2022 [37]	Results showed that the LR algorithm was the best algorithm to predict the mortality risk in patients with TBI in the emergency room triage setting.
Wolff, 2019 [31]	From the model viewpoint, their results showed successful experimentation of different ML techniques that have a recent interest in scientific research with great potential to be used as a triage decision support tool.
Wu, 2021 [50]	Compared to other clinical risk scores, their Least Absolute Shrinkage and Selection Operator (LASSO) regression model had a superior performance in predicting critical care outcomes in patients with chest pain and minimized the potential over-predicted and under-predicted critical care outcomes that could result in excessive resource allocation to low-risk patients and insufficient treatment of high-risk patients.
Xiao, 2023 [33]	Suggest that the proposed models significantly reduce the under-triage and over-triage issues compared to manual triage, outperform reference models, and can accurately predict patient severity level and clinical department.

Discussion

This manuscript used a systematic review process to explore various implementations of AI in emergency room triage care. The findings from this review show that AI may improve various aspects of medical care such as diagnosis, severity of condition, and urgency based on non-clinical findings. These findings are consistent with previous literature investigating the use of AI in medicine both in emergency and non-emergency settings to improve patient outcomes.

Overall, the outcomes of each AI implementation are measured consistently. Of the 29 articles in the review, 23 reported AUROC, and all stated the changes in the clinical practice of their AI. However, the scaling of data sets varied with 14 studies with a sample population of >100,000, six studies >10,000, seven studies > 1,000, and two studies <1,000. Additionally, the learning algorithms used in each varied with 19 studies comparing and contrasting an inconsistent combination of LR, random forest, decision tree, a genetic algorithm, deep neural network, and gradient boosting, as well as 10 implementing a single learning algorithm. Using AI in triage can produce significant changes to clinical practice, altering the way healthcare professionals manage patient care and prioritize resources in EDs. However, more research in this area is needed to ensure consistency with the scaling of data sets.

Hospital or intensive care unit admission

Eight studies examined the use of AI in determining the need to admit the patient to longer or more intensive care [23,24,30,34,42,44-46]. Six studies investigated hospital admission, while two examined intensive care unit admission. One of the difficulties with triage care is determining which patients will require more intensive care or monitoring. One of the common metrics for objective determination on whether to triage is via laboratory tests, vitals, patient history, and presenting symptoms [23,24,30,34,42,44-46]. These programs have the advantage of integrating all aspects of a presenting patient simultaneously to objectively determine a solution. Each system that was implemented was shown to improve the accuracy of predicting patient triage. Interestingly, the sole use of laboratory tests and demographic information was shown to have a favorable impact on triage predictability but to a lesser accuracy than its counterparts incorporating clinical data [36]. These results suggest that AI and ML may help transform the way EDs and intensive care units manage patient care, particularly in predicting which patients will need intensive care. By leveraging data-driven insights using AI, healthcare professionals can make more informed decisions, leading to better outcomes, optimized resource allocation, and improved patient safety.

The consistent superiority of ML models in determining and prioritizing patients within the ED setting holds the potential to redefine triage precision [23,24,30,34,42,44-46]. Reduced instances of under-triaging and over-triaging, coupled with the remarkable decrease in erroneous triage rates for critically ill patients, underscore the impact on the accuracy of patient prioritization [25,29]. This precision ensures that critical cases receive prompt attention, aligning with the essence of emergency care where timely interventions significantly influence outcomes. Rather than overtaking, AI may augment healthcare professional expertise to make more informed and consistent triage decisions to blend technological precision and human empathy.

Determination of critically Ill patients

Three studies examined the use of AI in identifying critically ill patients. These advancements showcase a high degree of accuracy and a substantial reduction in the time required to deliver critical services to patients in need [27-29]. Hwang et al.’s study focused on predicting interventions rather than triage accuracy and reported improved outcomes but acknowledged limitations such as being conducted in a single center, which may introduce selection bias, and the exclusion of patients receiving palliative care over intensive care [27]. Joseph et al.’s study investigated deep-learning approaches for identifying critically ill patients at ED triage and found that neural network and gradient-boosting models exhibited significantly higher accuracy compared to traditional triage methods [28]. However, the study acknowledged its limitations, including data being derived from a single hospital center with a higher population of critically ill patients, potential variability in ICU admission criteria across hospitals, and the possibility of mislabeling some critically ill patients who respond to ED treatment as not critically ill.

Furthermore, Liu et al.’s study explored the use of AI in determining triage features and emphasized the importance of the mode of arrival as a critical factor [29]. The model considered features, such as arrival time, arrival mode, and initial triage level, recognizing the potential variation of these factors across different geographic areas or facilities. The study also highlighted that age could be viewed as a continuous variable rather than a blunt cutoff and identified pulse pressure and shock index as beneficial indicators, surpassing the significance of systolic blood pressure (SBP) alone [29].

However, these studies' limitations include data being derived from a single hospital center with a higher population of critically ill patients, potential variability in ICU admission criteria across hospitals, and the possibility of mislabeling some critically ill patients who respond to ED treatment as not critically ill. More research may be needed to address these limitations, such as evaluating data from more than one institution and standardizing ICU admission criteria. The use of AI in identifying critically ill patients represents a paradigm shift in emergency medicine, offering transformative benefits for patient care and resource management in healthcare settings. This may include enhanced triage precision, improved clinical decision-making, streamlined workflow, reduced mortality and morbidity, proactive monitoring, and improved patient experience. By expediting the identification and treatment of critically ill patients, AI contributes to a more responsive and patient-centered healthcare experience, enhancing overall satisfaction. Moreover, these models may be beneficial in helping hospitals allocate resources such as ICU beds, staff, and medical equipment more efficiently, ensuring that critical cases receive timely care.

Predicting mortality

The incorporation of AI into ED settings for predicting mortality has yielded promising outcomes across ML models. Findings suggest there is potential for the XGBoost model to complement the current triage system and prevent under-triaging in critical cases [42]. ML algorithms for predicting mortality in patients with TBI provided evidence supporting clinical decision-making, but not without the common challenge of refining ML models for mortality prediction [26]. The use of an artificial neuronal network (ANN) for predicting the length of stay in syncope patients exhibited promise, with fair to good performance [48]. Moreover, the LR algorithm, despite lacking clinical laboratory data and imaging studies, outperformed other algorithms during initial triage for early mortality prediction [37]. These collective findings emphasize the ongoing research efforts needed to refine and enhance the accuracy and applicability of AI-based mortality prediction models in diverse ER settings [26,37,42,48]. Additionally, the overall success of each AI regardless of the training algorithm used showed improvements in predicting mortality. However, more research is needed.

The incorporation of AI into ED settings for predicting mortality has demonstrated considerable promise. ML models can improve the precision of triage, enabling early identification of high-risk patients and reducing human error. As AI technologies continue to evolve, their role in predicting mortality in ED settings will likely become even more crucial, paving the way for a more responsive and patient-centered approach to emergency care. The ongoing development and integration of AI into healthcare practices are expected to lead to continued improvements in patient outcomes and healthcare efficiency.

Clinical outcomes

Aside from improving ED workflows and determining urgent care for patients, AI can assist physicians by predicting the clinical outcomes for their patients [25,31,50]. ML in ED triage can enhance discriminative ability compared with conventional triage, showing high sensitivity for predicting critical care outcomes [25]. The implementation of these models could potentially reduce undertriage and prevent over-triage. ML models can be used as triage decision support tools in triage leading to better clinical outcomes [31]. The LASSO regression showed superior performance in predicting critical care outcomes, effectively minimizing potential over-predictions and under-predictions, and addressing concerns about resource allocation to low-risk patients and inadequate treatment for high-risk patients [50]. AI-based triage systems may facilitate better communication and coordination in the ED and with other departments by providing real-time data and insights. In the future, AI and ML algorithms could play a large role in improving clinical outcomes in ED triage. However, more research is warranted to explore these algorithms at multi-site hospitals in a variety of countries and explore the potential for selection bias. 

Operational efficiency

To reduce healthcare costs and to continue with the goal of AI to improve resource allocation, three studies examined staffing and other methods of directly improving operational efficiency, showcasing high accuracy levels across different triage levels, and reduction in the number of triage nurses at emergency triage stations [22,32,33]. AI algorithms could enhance operational efficiency by providing more accurate triage acuity assessments and significantly reduce under-triage and over-triage issues compared to manual triage [32,33]. Despite limitations in recognition accuracy and the inability to quantify resource allocation efficiency, the proposed models demonstrated the potential to enhance operational efficiency by accurately predicting patient severity levels and clinical departments [33]. While each study focused on a different aspect of management and operations, they all highlight the capacity for specific areas to be supplemented by AI. The use of data from a single institution and potential subjectivity in triage standards may lead to variability in results. More efforts are needed to test AI algorithms for their ability to enhance operational efficiency while checking for potential subjectivity in triage standards. 

The ability to accurately identify critical conditions, such as bacteremia, sepsis, and cardiopulmonary arrest, speaks to the transformative impact AI can have on patient outcomes [35,36,40,41]. AI may be a crucial mechanism for preventing adverse events in the ED waiting room, where the swift identification of patients at risk is paramount. It represents a leap toward ensuring patients receive timely attention and interventions even before stepping into the treatment rooms. Additionally, by providing timely and precise predictions, ML models empower healthcare professionals to prioritize and allocate resources efficiently, ensuring that critical cases receive prompt attention. Accurate predictions of hospital admission enable more efficient allocation of resources, including beds and personnel, based on anticipated patient needs. This not only streamlines ED operations but also contributes to improved bed management [48-50].

ED staff often need to conduct rapid assessments and decision-making processes. By leveraging ML algorithms, such as the XGBoost model with its commendable sensitivity, specificity, and overall prediction accuracy, the strain on medical staff can be mitigated. This is particularly crucial in high-pressure scenarios where prompt evaluation is essential for patient outcomes. ED crowding is an enduring challenge in healthcare, often leading to delays in patient care and increased stress among medical staff. The reviewed studies consistently suggest that AI has the potential to reduce these challenges by streamlining ED triage operations. The precise and rapid triage decisions offered by ML models have the potential to alleviate strain on healthcare professionals working in the crowded and dynamic environment of the ED [22,23].

Intervention

The XGBoost algorithm demonstrated high accuracy, with LR AUROC values ranging from 0.899 to 0.962 for interventions such as A-line insertion, oxygen therapy, high-flow nasal cannula, intubation, massive transfusion protocol, and inotropes [38]. The observed effect included a high degree of accuracy and reduced time needed to provide services to critical patients. Notably, the focus was on predicting specific interventions rather than triage accuracy. AUROC data for predicting outcomes such as tachycardia (0.836), hypotension (0.802), and hypoxia (0.802) using models that incorporated features from 15 minutes of passive monitoring were used [49]. The effect of this approach was improved discrimination over vital sign trends alone, suggesting the potential for enhancing the triage of initially stable patients at risk for decompensation. 

XGBoost algorithms have become prominent in emergency triage due to their effectiveness in prediction and intervention by improving triage processes in EDs. XGBoost is known for its high accuracy in predictive tasks. This high accuracy is crucial in emergency triage, where precise predictions can directly impact patient outcomes. It is fast and efficient, and speed is essential in EDs, where rapid decision-making can be a matter of life and death. It can handle complex data and derive meaningful insights to aid in identifying critical features that contribute to patient risk, aiding in more accurate triage decisions. The gradient boosting mechanism in XGBoost allows it to detect non-linear relationships in data, which is essential in ED triage, where patient conditions may involve complex interactions among various factors. Moreover, as EDs continue to collect more data, XGBoost models can be updated and refined, enabling continuous improvement in prediction accuracy. This adaptability allows EDs to stay current with evolving trends and practices.

Limitations of the articles in the review

The limited scope of the data may not fully capture the variability and complexity of patient populations and clinical practices in different healthcare environments [24,25,30,34,36,37,39,41-44,46,49]. The exclusion of ED visits with missing data during the preprocessing stage was noted as a significant limitation by researchers [30,34,41,43,44]. Moreover, one study only included patient history gathered from previous ED visits, while another omitted oxygen saturation level as a variable [23,44]. A study also pointed out the lower incidence of hypotension in comparison to other vital sign abnormalities impacting the performance of hypotension-prediction models [49]. This exclusion of a large amount of data could have potentially affected the generalizability of the model. Additionally, numerous datasets were derived from a single teaching hospital, also raising concerns about the generalizability of the prediction model to other hospital settings [22,24,26,28,29,31,33-36,38-42,45-47,49,50]. For instance, differences between rural and urban hospitals, private and public institutions, or community and university hospitals may affect patient profiles and therefore pose a challenge to implementing a standardized triage system.

All the authors of the articles in this review acknowledged that their study was based on retrospective data [22-50]. This absence of prospective validation is noteworthy since it may impact the applicability of the model to real-time clinical settings. There are limitations to the potential influence of the ML models on physicians’ behavior in a real-world scenario. In the event the ML model signals physicians to patients with possible critical outcomes, physicians may consider the model’s suggestion and accordingly adapt tests and interventions. This potential interaction between the predictive model and clinical decision-making could affect patient outcomes, representing a complex dynamic that the retrospective data may not fully capture. Chen et al.’s study mentions that retrospective data are susceptible to potential documentation errors, introducing a source of bias and inaccuracies into the dataset [34]. Chang et al.’s study highlighted that there are currently no established guidelines indicating the specific interventions needed for critically ill patients and the optimal timing for performing these interventions [38]. This lack of consensus underscores the need for standardization in critical care interventions, to ensure that patients receive the most effective and timely care. Therefore, caution should be exercised in applying the findings of this study to other healthcare settings, until further research and consensus are established.

When comparing the performance of ML models to that of healthcare professionals, Ivanov et al.’s study provided no account for individual nurse demographics such as years of nursing and triage experience. Although the nurses had standardized formal triage education, specific details such as the duration and frequency of nurse training were not available for analysis. This lack of information regarding nurse training and experience could have contributed to variability in the accuracy of the triage acuity assignments at the participating hospitals [32].

Limitations of the scoping review

The scoping review method used in this review is not without its limitations. The exclusion criteria based on titles and abstracts, such as inappropriate publication type or being beyond the scope of ED triage, rely on subjective judgments and may lead to inconsistencies in the selection process. The exclusion of studies based on elevated bias appraisal during the critical appraisal stage could result in the omission of valuable insights. Researchers should approach the findings with a critical awareness of these limitations and recognize their potential impact on the comprehensiveness, generalizability, and relevance of the synthesized evidence.

Implications for future research

The integration of AI in ED triage shows promise, but further studies are needed to address ethical considerations and ensure integration into clinical workflows. Future research could explore the long-term impact of AI on patient outcomes, the acceptance of AI among healthcare professionals, and the development of standardized guidelines for AI implementation in emergency care settings.

Ethical considerations surrounding the integration of AI in ED triage demand focused exploration. This includes issues related to patient privacy, informed consent, and the responsible use of patient data. Beyond ethical considerations, the acceptance and adoption of AI technologies by healthcare professionals are critical for successful implementation. Future research should explore the attitudes and concerns of emergency healthcare providers regarding the integration of AI in triage processes. Identifying barriers and facilitators to acceptance will inform strategies for effective implementation.

As AI continues to evolve in the field of emergency care, future research could focus on assessing the long-term impact of AI on patient outcomes. Investigation into the effectiveness of AI-driven triage models in improving patient outcomes, reducing mortality rates, and optimizing resource utilization will contribute essential evidence for their sustained integration into emergency healthcare settings.

## Conclusions

This scoping review included 29 articles to assess the use and potential impacts of AI and other forms of ML in the ED triage process. The findings consistently demonstrate the benefit of AI in improving triage efficiency and resource allocation, predicting hospital admissions, identifying critical conditions, and alleviating ED overflow and healthcare professional workload. As the field of AI in emergency medicine continues to evolve, this review may serve as a foundation for future research to explore ethical considerations, acceptance of AI among healthcare professionals, and long-term impact on patient outcomes. 
