# Correction: Use of Artificial Intelligence in Triage in Hospital Emergency Departments: A Scoping Review

**DOI:** 10.7759/cureus.c315

**Published:** 2025-09-24

**Authors:** Samantha Tyler, Matthew Olis, Nicole Aust, Love Patel, Leah Simon, Catherine Triantafyllidis, Vijay Patel, Dong Won Lee, Brendan Ginsberg, Hiba Ahmad, Robin J Jacobs

**Affiliations:** 1 Medicine, Dr. Kiran C. Patel College of Osteopathic Medicine, Nova Southeastern University, Fort Lauderdale, USA

This article has been corrected to fix the following typos in the 'Information Sources' paragraph:

- Total citations identified corrected from 1,142 to 1,140
- Articles remaining for further screening after removing duplicates corrected from 730 to 728

